# ConGPS: A Smart Container Positioning System Using Inertial Sensor and Electronic Map with Infrequent GPS

**DOI:** 10.3390/s23229198

**Published:** 2023-11-15

**Authors:** Shan Huang, Zihan Song, Hyung-Rim Choi, Jae-Joong Kim, Do-Myung Park, Byung-Kwon Park

**Affiliations:** Smart Logistics Research Center, Dong-A University, Busan 49315, Republic of Korea; 2270923@donga.ac.kr (S.H.); 1973741@donga.ac.kr (Z.S.); hrchoi@dau.ac.kr (H.-R.C.); jjkgb@dau.ac.kr (J.-J.K.); home21cc@gmail.com (D.-M.P.)

**Keywords:** GPS-denied area, multi-sensor technology, inertial navigation, real-time positioning, map-matching algorithm, smart container

## Abstract

Real-time global positioning is important for container-based logistics. However, a challenge in real-time global positioning arises from the frequency of both global positioning system (GPS) calls and GPS-denied environments during transportation. This paper proposes a novel system named ConGPS that integrates both inertial sensor and electronic map data. ConGPS estimates the speed and heading direction of a moving container based on the inertial sensor data, the container trajectory, and the speed limit information provided by an electronic map. The directional information from magnetometers, coupled with map-matching algorithms, is employed to compute container trajectories and current positions. ConGPS significantly reduces the frequency of GPS calls required to maintain an accurate current position. To evaluate the accuracy of the system, 280 min of driving data, covering a distance of 360 km, are collected. The results demonstrate that ConGPS can maintain positioning accuracy within a GPS-call interval of 15 min, even if using low-cost inertial sensors in GPS-denied environments.

## 1. Introduction

With the increasing complexity and scale of supply chains, the real-time visibility of container position is crucial in today’s global logistics industry [1,2,3]. It enhances supply chain coordination and minimizes operational risks such as theft or damage, as well as enabling efficient problem-solving in emergencies. A smart container is a container that is equipped with global positioning system (GPS) and a telecommunication system to send its position data to a remote server. The major technology currently employed for real-time positioning is GPS [4].

Although the widespread availability of precise positioning services provided by GPS can significantly enhance traceability in container transportation, GPS is subject to two limitations imposed during transportation: the battery life of the containers and the existence of GPS-denied areas [5]. Battery life is important because a battery cannot be replaced at the time of discharge during transportation. A GPS-denied area is where a satellite signal is obstructed or attenuated. For example, areas with dense buildings or long tunnels impede the seamless acquisition of GPS data and necessitate alternative positioning methods or hybrid positioning systems.

To address these limitations, various solutions have been proposed. As shown in Table 1, many researchers have sought to address the issue of positioning loss due to GPS-denied areas by employing inertial sensor data to predict trajectories [6,7,8,9]. This approach involves continuously tracking a vehicle’s position based on acceleration, direction, and attitude data provided by inertial sensors, assuming an initial position is available. Rogne et al. [10] applied strap-down inertial navigation technology to infer travel trajectories in GPS-denied areas. However, solutions including strap-down inertial navigation require high-precision inertial sensors and continuous calls. For container positioning systems, the cost associated with such a solution is prohibitively high. Furthermore, a high-precision inertial navigation system (INS) requires high-precision inertial measurement unit (IMU) sensors and high sampling frequency, which increase cost and power consumption.

Reducing the frequency of GPS calls to save power (e.g., by increasing the call interval to 1 h) introduces a significant issue of potentially missing important waypoint information. Typically, containers pass through or enter important intermediate stations/waypoints during transportation. If the GPS call frequency is too low, the container may fail to report those important events. A more innovative approach involves utilizing vehicle state data to predict vehicle location during GPS unavailability [9,16]. This approach primarily relies on state prediction through the application of Kalman filtering. Nevertheless, the system is susceptible to rapid error accumulation due to the utilization of only directional data as reference upon entering GPS-denied areas.

On the other hand, considerable research has been conducted to determine container positions through low-power Bluetooth/Wi-Fi/ZigBee connection technologies [11,12,13]. These technologies are viable alternatives for acquiring location data when GPS signals are unavailable. This approach is relevant within urban context monitoring scenarios. Nevertheless, it is imperative to acknowledge that integrating such alternative network connections engenders a heightened reliance upon existing network infrastructure.

An additional promising avenue pertains to visual simultaneous localization and mapping (SLAM), a technology that offers navigational and positional capabilities in contexts devoid of GPS signals [14,15]. Within GPS-denied areas, visual SLAM achieves navigation and localization through recognizing and tracking visual features such as key points and edges within the environment, coupled with an analysis of inter-frame motion relationships. This methodology operates independent of GPS signals, affording an accurate positioning service within GPS-denied settings such as indoor environments, urban canyons, and forested regions. Nonetheless, it is important to underscore that the processing demands of visual SLAM entail substantial computing resources, potentially constraining its applicability to the scenarios characterized by embedded devices with limited resource availability.

This study aimed to maintain real-time container positioning in GPS-denied areas while minimizing reliance on GPS calls. We propose a novel system named ConGPS that integrates inertial sensor data and electronic map data. ConGPS estimates the speed and heading direction of a moving container based on the inertial sensor data, the container trajectory, and the speed limit information provided by an electronic map. A container velocity estimation method was developed to estimate the current velocity based on the current heading direction, previous average speed, and map speed limit data. Furthermore, to enhance the precision of the position estimation, we introduced a map-matching algorithm. This algorithm aligns the estimated trajectory with the road network depicted on the electronic map to refine the position estimates. Through the results of a series of experiments, we demonstrate the superiority of the proposed algorithm in maintaining real-time position accurately while reducing the system’s dependence on GPS. In summary, the contributions of this research can be summarized as follows:

- Integrating the accelerometer and magnetometer data of low-cost inertial sensors with the speed limit data of electronic map for continuously predicting container velocity during inter-GPS calls or in GPS-denied environments.
- Devising a map-matching method utilizing the distance and direction similarity of container trajectory with the roads of electronic maps for adjusting container position.
- Evaluating performance via a field experiment over a 360 km course with 280 min of transportation.

The rest of this paper is organized as follows: proposing a positioning system in Section 2; analyzing accuracy in Section 3; A discussion in Section 4; concluding the paper in Section 5.

## 2. ConGPS

We propose a novel real-time container positioning system called ConGPS (a smart container positioning system using inertial sensor data and electronic map data with infrequent GPS). ConGPS can maintain positioning with infrequent GPS calls as well as in GPS-denied areas based on acceleration data, orientation data, and electronic map data.

### 2.1. Flowchart of ConGPS

**Figure 1:**
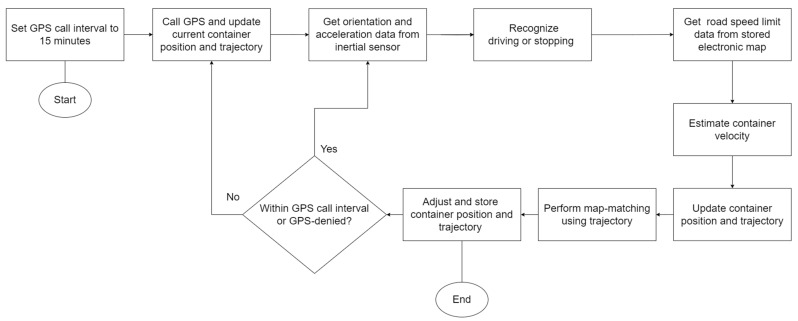
The flowchart of ConGPS.

Figure 1 depicts the flowchart of ConGPS. Firstly, the GPS call interval is set to 15 min. During this period, the container’s velocity estimation algorithm and the map-matching algorithm are continuously engaged to update the container’s position. After a GPS call and an update of container position and trajectory, the acceleration and orientation data are obtained from inertial sensors. The acceleration data are used to identify the current state of motion (Section 2.2.1). Based on the previous position, the speed limit data from the electronic map are retrieved to estimate the container speed (Section 2.2.2). The current position and trajectory are updated according to the estimated speed and direction. To reduce the estimation error, the current position and trajectory of the container are adjusted using the map-matching algorithm (Section 2.3). The above process is repeated during the GPS call interval. On reaching the interval, we call the GPS and correct the current position and trajectory in accordance with the GPS data.

### 2.2. Estimation of Container Positions using a Velocity-Estimating Algorithm

#### 2.2.1. Recognizing Container Motion State

Based on the acceleration data from an accelerometer, we can determine the current motion state of the container, i.e., whether it is in motion or at a standstill. As accelerometers are sensitive to various environmental influences and may produce noisy readings, it is essential to address these challenges to ensure the accuracy and reliability of the data. The simple moving average (SMA) method is employed to mitigate the impact of noise on the accelerometer data [17,18]. The SMA is defined as follows:

(1) $$SMA=\frac{\sum_{i=1}^{n}A_{i}}{n}$$

where n is window size; $A_{i}$ is the acceleration data in a single window *i*.

The window size is set to discriminate different movement states through experimentation and validation to achieve optimal performance. Based on the magnitude of the acceleration values, the container’s current state is classified as either moving or stationary. This binary classification provides a basis for subsequent modules in the system.

**Figure 2:**
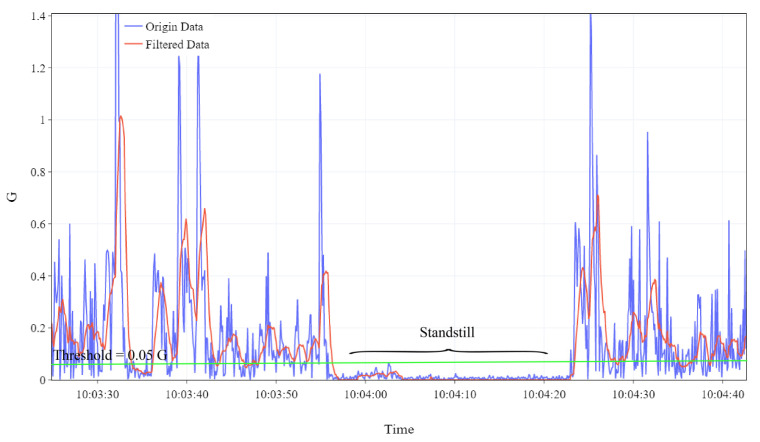
Acceleration data processed using the SMA.

Assuming that the y-axis of the accelerometer points to the direction of container’s forward movement, the filtered *y*-axis acceleration, after eliminating the effect of gravity, can effectively reflect the current motion state of the container. As illustrated in Figure 2, the acceleration threshold for movement state detection was set to 0.05 G (green line). When the consecutive acceleration readings for at least 3 s fall below the threshold, we assume that it is the noise induced by vibration and that the container is in a stationary state. Furthermore, in the ideal condition of uniform linear motion, the acceleration would theoretically be zero, excepting acceleration due to gravity. However, we assume that, in real transportation scenarios, containers experience minor acceleration and deceleration phases. Consequently, the proposed method is sufficiently robust to detect the container’s motion status.

#### 2.2.2. Estimating Velocity and Next Position of Container

Due to error accumulation, the accuracy of inertial navigation systems rapidly deteriorates within a few minutes [19,20]. This leads to the issue that a navigation system based on inertial sensors cannot be used for positioning over a long period. We propose a dynamic velocity estimation algorithm that incorporates electronic map data to address this issue, assuming the following throughout the transportation:

- The container is traveling only on the road.
- The traveling speed of the container remains relatively constant around the speed limit of the road.

We utilize the container’s stationary states as reference points. The phase between two consecutive stationary states encompasses the container’s acceleration, constant speed, and deceleration stages. We employ the road’s speed limit multiplied by a dynamic weight to estimate the container’s velocity. The algorithm can be summarized as follows:

(1) Initialization: Perform a GPS call to obtain the current position and retrieve the speed limit of the road segment on which the container is currently traveling.
(2) Retrieve the weight w and estimate the speed $V_{estimate}$ as the road’s speed limit times the weight w (in this study, let the initial weight be 0.75).
  (2) $$V_{estimate}=SpeedLimit\times w$$
(3) Read the orientation data from the magnetometer, HeadingAngle, which is the angle between the container’s current direction and true north. When the container faces true north, the heading angle is 0 degrees. For true east, it is 90 degrees; for true south, 180 degrees; and for true west, 270 degrees. Next, calculate NextPosition after Δt, which is the time interval between the current position and the next position. In this study, the frequency of calling the magnetometer was 1 Hz; therefore, Δt was equal to 1 s.
  (3) NextPosition=(PreviousLatitude+Δφ,PreviousLongitude+Δλ)
  (4) $$\Delta \lambda =\frac{\Delta x}{R\times \cos(PreviousLatitude)}$$
  (5) $$\Delta \varphi =\frac{\Delta y}{R}$$
  (6) $$\Delta x=V_{estimate}\times \cos(HeadingAngle)\times \Delta t$$
  (7) $$\Delta y=V_{estimate}\times \sin(HeadingAngle)\times \Delta t$$
  where Δφ the is amount of change in latitude; Δλ the is amount of change in longitude; Δx is the displacement in the x-direction (east–west) in meters; Δy is the displacement in the y-direction (north–south) in meters; R is the Earth’s radius, approximately 6371 km.
(4) Store HeadingAngle as the latest trajectory point.
(5) Iterate Step 2 to 4 until the next GPS call is made. Note that GPS calls are made in an interval (in this study, 15 min).
(6) After making the next GPS call, estimate the average GPS speed $V_{gps}$ from the distance $D_{gps}$ (estimated by the map-matching algorithm; see Section 2.3) divided by the interval time between the two GPS calls.
(7) Adjust the weight w.
  (8) $$w=\frac{v_{gps}}{v_{estimate}}$$
(8) Repeat Steps 2 to 7 until the container comes to a stop.
(9) Upon container stoppage, store w. When the container resumes travel, retrieve w for subsequent calculations.

### 2.3. Adjustment of Container Positions using Trajectory-Based Map-Matching Algorithm

A trajectory-based map-matching algorithm is a method aimed at associating trajectory data with the geographical road network [21,22]. It facilitates the alignment of continuous spatiotemporal location points, constituting a trajectory, to the corresponding road segments represented in digital maps. Compared to the conventional distance-based approaches [23], a trajectory-based map-matching algorithm determines the precise path traversed by containers by matching trajectory points to the road network topology. Python’s OSMnx library [24] can be used to obtain offline map data from OpenStreetMap [25].

Our trajectory-based map-matching algorithm is composed of three subalgorithms. The first involves distance-similarity computing, the second involves direction-similarity computing, and the final involves next-position adjustment. These algorithms can be summarized as follows:

#### 2.3.1. Distance-Similarity Computing

**Figure 3:**
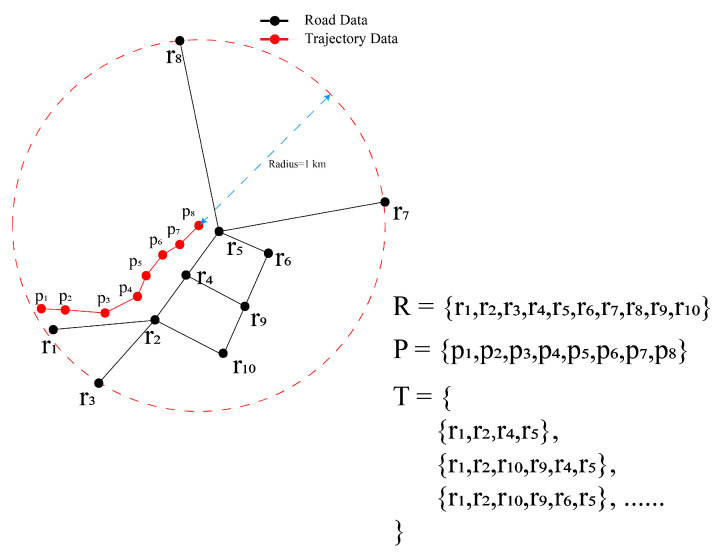
Example of R, P, and T.

**Figure 4:**
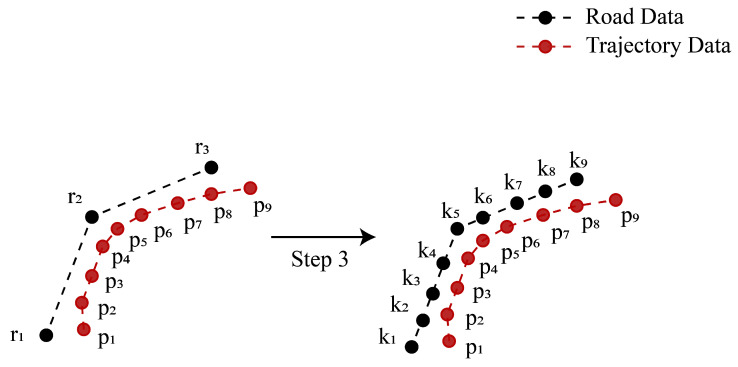
Example of dividing path data based on the number of trajectory points.

(1) Acquire road data $R=\left\{r_{i},i\in N^{*}\right\}$ and trajectory data $P=\left\{p_{i},i\in N^{*}\right\}$, where $r_{i}$ and $p_{i}$ are composed of the latitude and longitude coordinates, respectively. R refers to all roads within 1 km of the current position. P is a series of location points produced by ConGPS within 1 km of the current position. Figure 3 illustrates an example data structure. It is noteworthy that R is sparser than P because road data typically only consist of endpoints, turning points, and intersections of roads, while trajectory data comprise a series of closely spaced waypoints.
(2) Compose a set of paths T, as shown in Figure 3. A path in T comprises the road data points such that the start point is within 300 m of the start point of P, and the end point is within 300 m of the end point of P. In Figure 3, the start point is $r_{1}$, and the end point is $r_{5}$ in a relevant path. We can use the breadth-first search algorithm [26] to obtain T.
(3) For each path in T, the path is evenly divided to have the same size as that of P, resulting in a set $K=\left\{k_{i},i\leq \left|P\right|\right\}$. This process aims to transform the sparse road data into a format that allows for comparison with P. As shown in Figure 4, P contains 9 points and the path $\left\{r_{1},r_{2},r_{3}\right\}$ in T contains 3 points (representing the start, turning, and end of the road). Then, to compare the distance similarity between roads and trajectories, the path needs to be evenly divided into 9 points, as shown in Figure 4.
(4) Calculate the distance between K and P using the Haversine formula [27]. The computation formula is as follows:
  (9) $$d=2\times arctan(\;\sqrt{a},\;\sqrt{1-a})\cdot R$$
  (10) $$a=sin²\left(\Delta \varphi /2\right)+cos\;\varphi_{1}\times cos\;\varphi_{2}\times sin^{2}\left(\Delta \lambda /2\right)$$
  where d is the great-circle distance between two points; $\varphi_{1}$ and $\varphi_{2}$ are the latitudes of two points; Δφ is the difference in latitude; Δλ is the difference in longitude; R is the Earth’s radius (mean radius = 6371 km).
(5) Find all d for each K, resulting in the distance similarity denoted as $D=\left\{d_{i},i\leq \left|T\right|\right\}$.

#### 2.3.2. Direction-Similarity Computing

**Figure 5:**
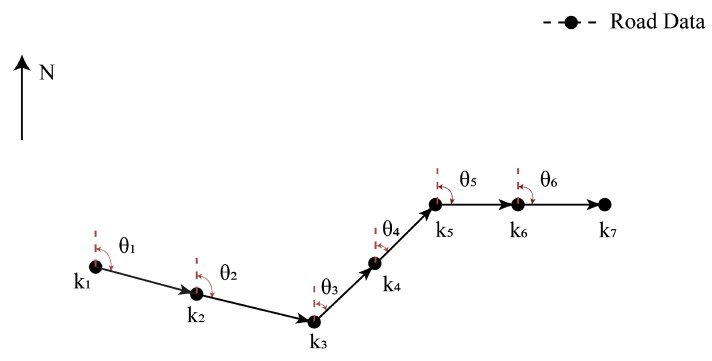
Example of heading angle representations.

(1) to (3) The same as for distance-similarity computing.
(4) The elements in K are sequentially paired and transformed into vectors. As shown in Figure 5, assuming that K contains 7 points, the paired vectors are $\left\{\vec{k_{1}k_{2}},\vec{k_{2}k_{3}},\vec{k_{3}k_{4}},\vec{k_{4}k_{5}},\vec{k_{5}k_{6}},\vec{k_{6}k_{7}}\right\}$. The HeadingAngle of each vector is then computed, resulting in a set $V=\left\{\theta_{i},i\leq \left|K\right|-1\right\}$. We define HeadingAngle as the angle between the vector and the true north as follows:
  (11) HeadingAngle=arctan(x,y)
  (12) $$x=\cos(\varphi_{b})\times \sin(\Delta \lambda )$$
  (13) $$y=\cos(\varphi_{a})\times \sin(\varphi_{b})-\sin(\varphi_{a})\times \cos(\varphi_{b})\times \cos(\Delta \lambda )$$
  where a and b are two points of the vector; φ is latitude; λ is longitude.
(5) Apply Step 4 to P in the same way as K, obtaining $Q=\left\{\theta_{i},i\leq \left|P\right|-1\right\}$.
(6) Compute the Pearson correlation coefficient ρ for V and Q.
  (14) $$\rho_{}=\frac{\mathrm{cov}(V,Q)}{\sigma_{V}\sigma_{Q}}$$
  where $\sigma_{V}$ is the standard deviation of V; $\sigma_{Q}$ is the standard deviation of Q.
(7) For each K, repeat Steps 4 to 6, resulting in the direction similarity denoted as $W=\left\{\rho_{i},i\leq \left|T\right|\right\}$.

#### 2.3.3. Next-Position Adjustment

(1) We compute trajectory similarity $s_{i}$ for each K as follows:
  (15) $$s_{i}=\rho_{i}-k\times d_{i}$$
  where $d_{i}$ is the distance similarity for each K; $\rho_{i}$ is the direction similarity for each K; k is the weight used to balance the direction similarity and distance similarity (in this study, k was set to 0.5).
(2) We select K having maximum $s_{i}$ as the matching path with P. After finding the matching path, we adjust the current position to the point in the matching path that has the shortest distance to the estimated position using the method in Section 2.2.

## 3. Experimental Evaluation

We employed two Android smartphones of an identical model to collect data about vehicular movement. Table 2 presents the relevant parameters of the inertial sensors installed in the smartphones. One smartphone was used to collect GPS data at a frequency of 1 Hz, thereby delineating an accurate representation of the authentic trajectory. The other smartphone was used to capture acceleration data at a rate of 10 Hz and magnetometer data at a rate of 1 Hz. The dataset encompassed a comprehensive temporal span of 360 min (337 min of travel time and 23 min of stop time) over a total vehicular travel distance of 280 km. Preceding each instance of data collection, a calibration procedure of the smartphone’s magnetometer was conducted at a locale devoid of pronounced magnetic field interferences.

**Figure 6:**
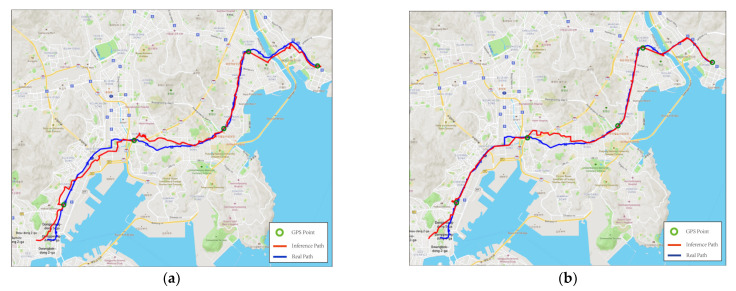
(**a**) Trajectory using the velocity-estimating algorithm only. (**b**) Trajectory using both the velocity-estimating and map-matching algorithm.

Figure 6 illustrates the practical performance of the proposed algorithm along an exemplary trajectory. The container travels from the upper-right corner to the lower-left corner of the map. Figure 6a depicts the trajectory obtained only through the velocity estimation algorithm in Section 2.2. Figure 6b depicts the trajectory adjusted after undergoing the map-matching algorithm in Section 2.3, resulting in the path better aligned with road on which the container is traveling. The green dots denote the positions corrected with GPS calls with a time interval of 15 min. In Figure 6a, although the trajectory is similar to the road in direction, the trajectory travels outside the road due to the limited accuracy of the magnetometer and the algorithm. In contrast, the trajectory in Figure 6b is located inside the road owing to the adjustment produced by the map-matching algorithm.

**Figure 7:**
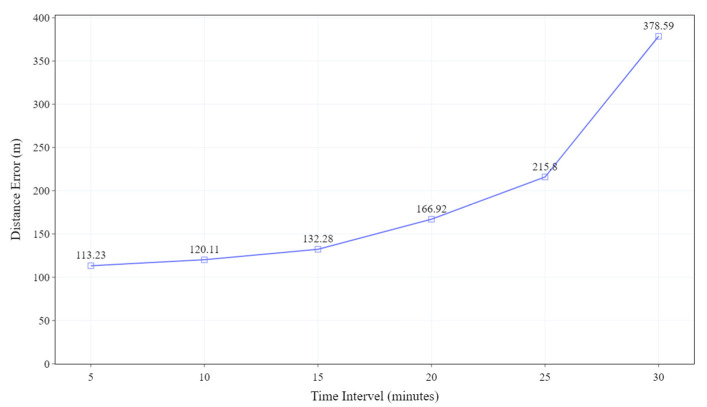
The average distance error between the estimated trajectory and the real trajectory along the GPS call interval.

On the other hand, the errors appearing in the middle and in the end in Figure 6b are caused by the accumulation of acceleration error and magnetometer error. The errors are regularly corrected at each GPS point. Figure 7 illustrates the average distance error of the trajectory generated by the proposed algorithm under varying intervals of GPS call. The average distance error of the trajectory is the average of all the distances between each pair of points (one on the trajectory and the other on the road). The distance is calculated according to the Haversine formula [27]. The average distance error at the 5 min, 10 min, and 15 min intervals increases slowly. However, after the 15 min interval, the error increases rapidly. Specifically, at the 30 min interval, the average distance error increases to 2.85 times greater than that at the 15 min interval.

**Figure 8:**
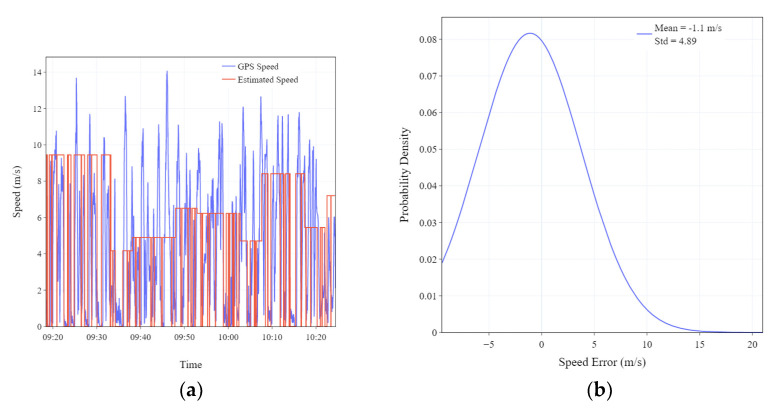
(**a**) Comparison between GPS speed ($V_{\mathrm{gps}}$) and estimated speed ($V_{estimate}$). (**b**) The probability density distribution of the error between $V_{\mathrm{gps}}$ and $V_{estimate}$.

In addition, we evaluated the performance of the motion status detection and velocity estimation algorithm in Section 2.2. Figure 8a compares $V_{\mathrm{gps}}$ and $V_{estimate}$ along an exemplar trajectory. $V_{\mathrm{gps}}$ is computed from GPS data sampled at 1 Hz. $V_{estimate}$ is derived through the velocity estimation algorithm and the container motion status recognition algorithm in Section 2.2. The trend of change in $V_{estimate}$ is similar to that in $V_{\mathrm{gps}}$. Figure 8b shows the distribution of error between $V_{estimate}$ and $V_{\mathrm{gps}}$ in the dataset. The average discrepancy between $V_{estimate}$ and $V_{\mathrm{gps}}$ rests at approximately −1 m per second, showing that $V_{estimate}$ effectively corresponds to $V_{\mathrm{gps}}$.

**Figure 9:**
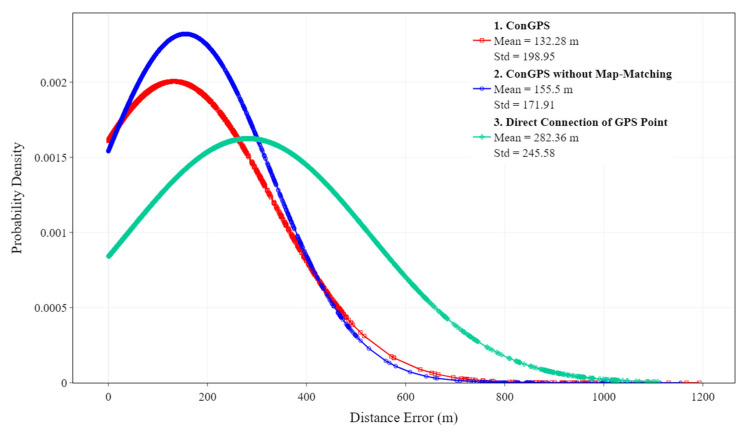
The probability density distribution of distance errors between the estimated trajectories and the actual trajectory.

Figure 9 presents the probability density distribution of distance errors between the estimated trajectories and the actual trajectory in 15 min intervals. Curve 1 corresponds to the error between the trajectory obtained through ConGPS and the actual trajectory. Curve 2 corresponds to the error when the trajectory was obtained through ConGPS without the map-matching algorithm. Curve 3 corresponds to the error when the trajectory was obtained through a direct connection of GPS points. Curve 1 exhibits the most favorable performance, with an average distance error of 132.28 m. This implies that, in comparison to the trajectory obtained via directly connecting GPS points, the proposed algorithm enhances the localization accuracy by approximately twofold. On the other hand, the average distance error of curve 2 is 1.12 times that of curve 1, which illustrates the improvement in the map-matching algorithm on positioning accuracy.

## 4. Discussion

In this study, we developed a container positioning system that integrates both inertial sensor data and electronic map data. Our findings illustrate that the incorporation of road data from electronic maps enables an accurate prediction of container trajectory, employing low-precision accelerometers and magnetometers. We observed that the proposed system can maintain positioning accuracy up to a 15 min interval of GPS calls with an average distance error of 132.28 m. Due to the relatively small difference in distance error and to minimize the frequency of GPS calls, we employed a 15 min interval to assess the performance of the proposed system.

The performance partially stems from the dynamic adjustment of the estimated velocity based on the utilization of the road speed limit data obtained from electronic maps, resulting in an average speed error of approximately 1 m per second, as shown in Figure 8b. It is noteworthy that the algorithm circumvents the error accumulation inherent to traditional INS algorithms, thereby averting significant deviation between estimated and actual speed. In addition, the use of accelerometer data for detecting container motion states also contributes to avoiding error accumulation. In particular, the proposed algorithm obviates the necessity for high-precision sensors and the acquisition of supplementary sensor data.

Compared to prior research, this study’s novelty resides in utilizing speed limit data and trajectory data to estimate container positions during GPS-denied periods. Sun et al. [16] proposed a navigation solution for GPS-absent scenarios, reporting errors below 100 m within 60 min. However, this solution necessitates employing relatively sophisticated onboard INS equipment and is confined to a singular testing route. Chunhakam et al. [9] introduced a navigation algorithm employing magnetometers and speed data retrieved via truck on-board diagnostics (OBD), yielding an evaluated error of 30 m. The reliance on speed information obtained exclusively from the truck’s OBD system limits their method’s applicability. In contrast, our work integrates information such as road speed limits and road trajectories with inertial sensor data, enhancing accuracy during GPS-denied periods. Furthermore, it reduces the frequency of GPS calls required while upholding positioning accuracy.

There are some limitations worth mentioning. As the GPS call interval increases, the proposed system’s distance error also escalates. This is attributed to the simplicity of the velocity estimation algorithm. Future research endeavors may therefore contemplate refining the velocity estimation algorithm amalgamated with road and traffic information. For instance, incorporating intersections and road congestion levels could elevate the positioning accuracy.

ConGPS can provide a real-time positioning service for moving containers. The algorithm for recognizing container motion state just scans the input data to calculate the SMA and recognize a stationary or moving state, so that its time complexity is *O*(*n*), where *n* is the input data size. The algorithm for estimating the velocity and next position of a container scans the magnetometer data and calculates the velocity and the next position, so that its time complexity is also *O*(*n*). The algorithm for distance-similarity computing scans the road data and the trajectory data, making all possible paths from the road data to compute the distance between the path and the trajectory. When there are *n* points in the road data, maximal *n!* paths should be considered, so that the time complexity in making all possible paths is *O*(*n!*). Therefore, we should control the road data size to prohibit the path-making time from growing exponentially. In this study, we retrieved the road data only from within 1 km of the current position. Furthermore, we considered only those paths where the start point was within 300 m of the start point of the trajectory and where the end point was within 300 m of the end point of the trajectory. The algorithm for direction-similarity computing sequentially scans the path data to compute the heading angles, so that its time complexity is *O*(*n*), where *n* is the number of points in the path. Overall, we can control the time complexity of the proposed algorithm to be *O*(*n*) so as to provide a real-time positioning service for a moving container.

## 5. Conclusions

In this study, we developed a smart container positioning system named ConGPS that combines inertial sensor data and electronic map information. ConGPS innovatively leverages road trajectory data and road speed limit data from electronic maps as well as inertial sensor data to achieve accurate positioning with reduced GPS calls. ConGPS also cost-effectively furnishes a real-time positioning service with low-precision inertial sensors.

The limitation of this study is that the distance error of ConGPS starts to increase rapidly when the GPS request interval exceeds 15 min. This is attributed to the simplicity of the velocity estimation algorithm. Subsequent research endeavors could consider incorporating additional road- or traffic-related data to reduce distance error. For instance, such data as traffic congestion levels, road construction information, and the number of available lanes could be explored to refine the velocity estimation.

## Figures and Tables

**Table 1:** Comparison of techniques for container positioning.

Works	Features	Advantage	Limitation
[6,7,8,9]	INS, GPS	Precise position	INS can only maintain short-range position guessing
[11,12,13]	Low-power WIFI, Bluetooth, and ZigBee	Low battery consumption, accurate position	Need to connect to edge nodes
[14,15]	SLAM	No additional infrastructure required	Requires substantial allocation of computational resources

**Table 2:** Sensor specifications.

Sensors	Range	Resolution
Accelerometer	±16 g	0.5 mg/LSB
Magnetometer	±2 Gauss	0.0667 mG/LSB
GPS	<2.5 m	<0.1 m/s

## Data Availability

The data presented in this study are available on request from the corresponding author. The data are not publicly available due to privacy and confidentiality concerns.

## References

[1] Salah K., Alfalasi A., Alfalasi M., Alharmoudi M., Alzaabi M., Alzyeodi A., Ahmad R.W. IoT-Enabled Shipping Container with Environmental Monitoring and Location Tracking. Proceedings of the 2020 IEEE 17th Annual Consumer Communications & Networking Conference (CCNC).

[2] Mahmood S., Hasan R., Ullah A., Sarker K.U. SMART Security Alert System for Monitoring and Controlling Container Transportation. Proceedings of the 2019 4th MEC International Conference on Big Data and Smart City (ICBDSC).

[3] Chan A.S., Sutapa I.N. Truck management integrated information system in a shipping line company. Proceedings of the 2017 International Conference on Soft Computing, Intelligent System and Information Technology (ICSIIT).

[4] Wang Y., Ning X., Xu X. (2023). An Improved In-Motion Coarse Alignment Method for SINS/GPS Integration with Initial Velocity Error Suppression. Sensors.

[5] Li X., Zhang X., Chen K., Feng S. Measurement and analysis of energy consumption on Android smartphones. Proceedings of the 2014 4th IEEE International Conference on Information Science and Technology.

[6] Ibrahim A., Abosekeen A., Azouz A., Noureldin A. (2023). Enhanced Autonomous Vehicle Positioning Using a Loosely Coupled INS/GNSS-Based Invariant-EKF Integration. Sensors.

[7] Cao Y., Bai H., Jin K., Zou G. (2023). An GNSS/INS Integrated Navigation Algorithm Based on PSO-LSTM in Satellite Rejection. Electronics.

[8] Davidson P., Vazquez M.A., Piche R. Uninterrupted portable car navigation system using GPS, map and inertial sensors data. Proceedings of the 2009 IEEE 13th International Symposium on Consumer Electronics.

[9] Chunhakam P., Pummarin P., Jeen-Im P., Wardkien P., Wisartpong P., Lertteerada K. GPS Positon Predicting System by Kalman Filter with Velocity from OBD and Direction from Magnetometer. Proceedings of the 2021 9th International Electrical Engineering Congress (iEECON).

[10] Rogne R.H., Bryne T.H., Fossen T.I., Johansen T.A. (2021). On the Usage of Low-Cost MEMS Sensors, Strapdown Inertial Navigation, and Nonlinear Estimation Techniques in Dynamic Positioning. IEEE J. Ocean. Eng..

[11] Ni P. (2021). Intelligent Containers for the Goods Transport. Proceedings of the 2021 4th International Conference on Information Systems and Computer Aided Education.

[12] Ungurean I., Chi J., Wang K., Gaitan N.C., Yao H., Yang Y. Mobile ZigBee Network in a High RF Interference Environment. Proceedings of the 2019 International Conference on Sensing and Instrumentation in IoT Era (ISSI).

[13] Talukder N., Ahamed S.I., Abid R.M. Smart Tracker: Light Weight Infrastructure-less Assets Tracking solution for Ubiquitous Computing Environment. Proceedings of the 2007 Fourth Annual International Conference on Mobile and Ubiquitous Systems: Networking & Services (MobiQuitous).

[14] Luo F., Liu Z., Zou F., Liu M., Cheng Y., Li X. (2023). Robust Localization of Industrial Park UGV and Prior Map Maintenance. Sensors.

[15] Wells L.A., Chung W. (2023). Vision-Aided Localization and Mapping in Forested Environments Using Stereo Images. Sensors.

[16] Sun C., Li K. (2023). A Vehicle-Carried INS Positioning Accuracy Improvement Method by Using Lateral Constraint in GPS-Denied Environment. IEEE Trans. Veh. Technol..

[17] Moving Average. https://en.wikipedia.org/wiki/Moving_average.

[18] Song Z., Park H.-J., Thapa N., Yang J.-G., Harada K., Lee S., Shimada H., Park H., Park B.-K. (2022). Carrying Position-Independent Ensemble Machine Learning Step-Counting Algorithm for Smartphones. Sensors.

[19] Chen C., Lu X., Markham A., Trigoni N. (2018). IONet: Learning to Cure the Curse of Drift in Inertial Odometry. Proc. AAAI Conf. Artif. Intell..

[20] El-Sheimy N., Hou H., Niu X. (2008). Analysis and Modeling of Inertial Sensors Using Allan Variance. IEEE Trans. Instrum. Meas..

[21] Chawathe S.S. Segment-Based Map Matching. Proceedings of the 2007 IEEE Intelligent Vehicles Symposium.

[22] Hashemi M., Karimi H.A. (2016). A weight-based map-matching algorithm for vehicle navigation in complex urban networks. J. Intell. Transp. Syst..

[23] Dakai Y., Baigen C., Yifang Y. An improved map-matching algorithm used in vehicle navigation system. Proceedings of the 2003 IEEE International Conference on Intelligent Transportation Systems.

[24] Boeing G. (2017). OSMnx: New methods for acquiring, constructing, analyzing, and visualizing complex street networks. Comput. Environ. Urban Syst..

[25] OpenStreetMap Planet Dump. https://www.openstreetmap.org/.

[26] Breadth-First Search. https://en.wikipedia.org/wiki/Breadth-first_search.

[27] Haversine Formula. https://en.wikipedia.org/wiki/Haversine_formula.

